# Sensitivity and specificity of automated blood pressure devices to detect atrial fibrillation: A systematic review and meta-analysis of diagnostic accuracy

**DOI:** 10.3389/fcvm.2022.956542

**Published:** 2022-08-12

**Authors:** Edmond W. L. Tang, Benjamin H. K. Yip, Chun-Pong Yu, Samuel Y. S. Wong, Eric K. P. Lee

**Affiliations:** ^1^Jockey Club School of Public Health and Primary Care, Faculty of Medicine, The Chinese University of Hong Kong, Shatin, Hong Kong SAR, China; ^2^Li Ping Medical Library, The Chinese University of Hong Kong, Shatin, Hong Kong SAR, China

**Keywords:** atrial fibrillation, blood pressure, hypertension, electrocardiogram (ECG), sensitivity and specificity, meta-analysis

## Abstract

**Background:**

Atrial fibrillation (AF) is a prevalent and preventable cause of stroke and mortality.

**Aim:**

This systematic review and meta-analysis aimed to investigate the sensitivity and specificity of office and out-of-office automated blood pressure (BP) devices to detect AF.

**Methods:**

Diagnostic studies, extracted from databases such as Ovid Medline and Embase, on AF detection by BP device(s), electrocardiography, and reported sensitivity and specificity, were included. Screening of abstracts and full texts, data extraction, and quality assessment were conducted independently by two investigators using Covidence software. The sensitivity and specificity of the BP devices were pooled using a random-effects model.

**Results:**

Sixteen studies including 10,158 participants were included. Only a few studies were conducted in primary care (*n* = 3) or with a low risk of bias (*n* = 5). Office BP devices, which utilised different algorithms to detect AF, had a sensitivity and specificity of 96.2 and 94%, respectively. Specificity was reduced when only one positive result was considered among consecutive BP measurements. Only a few studies (*n* = 3) investigated out-of-office BP. Only one study (*n* = 100) suggested the use of ≥79 and ≥26% of positive readings on 24-h ambulatory BP measurements to detect AF and paroxysmal AF, respectively.

**Conclusions:**

Office BP devices can be used clinically to screen for AF in high-risk populations. Clinical trials are needed to determine the effect of AF screening using office BP devices in reducing stroke risk and mortality. Further studies are also required to guide out-of-office use of BP devices for detecting paroxysmal AF or AF.

**Systematic review registration:**

https://www.crd.york.ac.uk/prospero/display_record.php?ID=CRD42022319541, PROSPERO CRD42022319541.

## Introduction

Atrial fibrillation (AF) is an independent and preventable cause of stroke; patients with AF have a more than twofold increased stroke risk (1). Furthermore, AF is associated with systematic embolism, dementia, and mortality (2, 3). The prevalence of AF increases with age, affecting 17.8% of adults aged ≥ 85 years (4). Despite the associated complications, AF is consistently under diagnosed due to frequent asymptomatic occurrences (5). Once diagnosed, AF-related stroke and death can be effectively prevented by administration of oral anticoagulants in high-risk patients (6, 7), and hence, most international guidelines recommend regular screening of individuals aged > 65 years for AF (8–10). Preliminary evidence suggests that AF screening enhances detection rates and increases anticoagulant prescriptions (11, 12).

Ironically, the best AF screening method is still unknown. Although international guidelines suggest the application of opportunistic pulse palpation or 12-lead electrocardiogram (ECG) for AF screening, pulse palpation is only modestly accurate (sensitivity, 91%; specificity, 74%). Furthermore, pulse palpation was most commonly conducted during manual BP measurements using a mercury or aneroid device, which have been progressively replaced by automated devices (13, 14). The 12-lead ECGs interpreted by cardiologists remain the reference standard to diagnose AF; however, systematic use of this method can be costly and may burden healthcare systems (14). In the past two decades, various automated blood pressure (BP) devices have incorporated irregular heartbeat detectors or AF detectors, allowing affordable and feasible AF screening (15). As most international guidelines recommend regular screening of hypertension (HT) in adults, these automated BP machines can simultaneously screen for AF (16). The use of BP devices may also automatically target high-risk populations because up to 90% of patients with AF also experience HT (14).

A high-quality diagnostic meta-analysis on AF screening using automated BP devices is lacking. Previous meta-analyses have had the following limitations: (i) conducted using only a few studies (as few as 4), (ii) investigated only Microlife devices, (iii) did not investigate different AF detection algorithms, (iv) did not discuss out-of-office BP devices, (v) did not conduct relevant subgroup/sensitivity analyses despite high heterogeneity in the results, or (vi) meta-analysed sensitivity and specificity data separately (which is not recommended because sensitivity and specificity are often correlated) (14, 17–21). The National Institute for Health and Clinical excellence (NICE) guidelines validated and recommended only the Watch BP Home A (Microlife AF, Switzerland) automated BP machine (22). In contrast to the Food and Drugs Administration (FDA)-approved AF detector used by Watch BP, other BP devices use irregular heartbeat monitors, which are not FDA-approved and may use different algorithms which were not described in previous systematic reviews (14). Hence, it is not known whether these automated BP machines have similar accuracy. Furthermore, although out-of-office BP measurements (including 24-h ambulatory BP monitoring [ABPM] and home BP monitoring [HBPM]) are increasingly recommended by international guidelines to diagnose and manage HT, their sensitivity and specificity to detect AF have not been adequately analysed by previous reviews. Repeated BP measurements during HBPM or ABPM allows detection of AF as well as paroxysmal AF (pAF); pAF carries comparable stroke risk to persistent AF (14, 23). The latest meta-analysis used single-lead ECG as the reference standard; however, single-lead ECG has a sensitivity and specificity of 93.9 and 90.1%, respectively, of detecting AF when compared to a 12-lead ECG, and is therefore not recommended (17, 24).

This study aimed to investigate the sensitivity and specificity of automated BP devices in detecting AF. Whenever feasible, subgroup analyses (e.g., office BP vs. HBPM/ABPM and between different BP devices) were conducted. In addition to 12-lead ECG, continuous ECG monitoring techniques (such as 24-h Holter monitoring) were included in studies investigating AF detection by ABPM or HBPM (12, 25). To summarise into the Participants, Index test, Reference standards and Outcomes (PIRO) format: *P* = adults with or without diagnosis of HT; I = detection of AF by automated BP devices; R = 12-lead ECG (or other continuous ECG methods for HBPM/ABPM); O = sensitivity and specificity to detect AF.

## Methods

This study was registered on PROSPERO (no.: CRD42022319541).

### Study eligibility

All diagnostic and observational studies in which the same participants, who were at least 18 years of age (with or without HT), underwent AF detection using automated BP device(s) and 12-lead ECG (or continuous ECG technique [for ABPM/HBPM studies]) were included. Further, studies that reported sensitivity and specificity (or equivalent raw data), and those published in English were included. Studies on pregnant or paediatric populations were excluded due to associated increased sinus arrhythmias (26). Animal studies, commentaries, and reviews were also excluded. As abstracts presented at major HT conferences were published in international peer-reviewed journals, the search strategies were able to find studies that had not been published. When only abstracts were available, the authors were contacted for any published reports or articles.

### Search strategy

The databases Ovid Medline, Embase, Scopus, CINAHL complete, and Web of Science were searched from inception until March 2, 2022. Keywords such as “ambulatory blood pressure”, “automated office blood pressure”, “automated oscillometric blood pressure, blood pressure monitor, blood pressure device, and atrial fibrillation” were used as search terms. The search was limited to studies in English and those involving only adults. The detailed search strategies used for these databases are presented in Supplementary Table S1.

### Study screening and data extraction

Studies resulting from the search were imported into the Covidence program (Covidence systematic review software, Veritas Health Innovation, Melbourne, Australia; available at www.covidence.org). Two investigators (EKP and TWL) independently assessed the eligibility of the studies by screening the titles and abstracts, and subsequently the full texts in the Covidence program. Authors were contacted to assess the possibility of duplicated data when suspected. Data were extracted independently by two investigators (EKP and TWL). Any differences between the eligibility assessment and data extraction were resolved by consensus.

The following data were extracted from the included studies: (i) study characteristics (e.g., number of participants, conducted in primary care or specialist centre, and funding source); (ii) participants” demographic details (age, sex, and comorbidities); (iii) details of automated BP measurement methods (ABPM/HBPM/office BP, device model, number of measurements, algorithms to detect AF); (iv) details of reference standard (12-lead ECG or continuous ECG methods), and (v) sensitivity/specificity of automated BP devices to detect AF.

### Quality assessment

The quality assessment was based on QUADAS-2, which was developed for systematic reviews of diagnostic accuracy studies (27). Under the domain “reference test,” we considered the risk-of-bias to be low if the ECGs were interpreted by cardiologists because general practitioners have lower sensitivity to detect AF on ECGs (14). Under the domain “flow and timing,” we considered the risk-of-bias to be low if the index tests were carried out immediately before or after or simultaneously with the reference tests because AF can self-terminate (i.e., paroxysmal AF). The study was at low risk of bias only when all signalling questions were not concerned, while all other studies were categorised under unknown risk or high risk of bias. Quality assessments were independently conducted by EKP and TWL.

### Analysis

All meta-analyses were conducted using Stata software (StataCorp. 2021. Stata Statistical Software: Release 17. College Station, TX, StataCorp LLC) unless specified. Sensitivity and specificity of automated BP devices were pooled using the “metandi” function and a random effect model (this model was used because studies were conducted in different populations with different models of automated BP devices). Subgroup analyses were conducted for (i) office BP versus out-of-office BP, (ii) number of BP measurements to detect AF, and (iii) devices from different manufacturers. Sensitivity analyses were conducted with (i) only studies with a low risk of bias, (ii) only BP devices that were found to be validated for BP measurements (as listed on www.stridebp.org), (iii) studies with a higher prevalence of AF (at ≥15 and 20%), (iv) studies conducted in specialist centres, and (v) studies receiving funding from BP device manufacturers. Microlife devices (which is the preferred device by the NICE guideline) were used in the main analysis because some included studies compared Microlife with non-Microlife devices. Heterogeneity was assessed by visual inspection of the scatter plot in the receiver operating characteristic (ROC) plane, and calculation of the area of the 95% prediction ellipse and median odds ratio (MOR; possible values of median odds ratio ranged from 1 [no heterogeneity] to infinity [high heterogeneity]) (28). Calculation of area of the 95% prediction ellipse and MOR were conducted using R (R: A language and environment for statistical computing. R Foundation for Statistical Computing, Vienna, Austria.) and specific formula respectively (28).

## Results

### Characteristics of included studies

**Figure 1:**
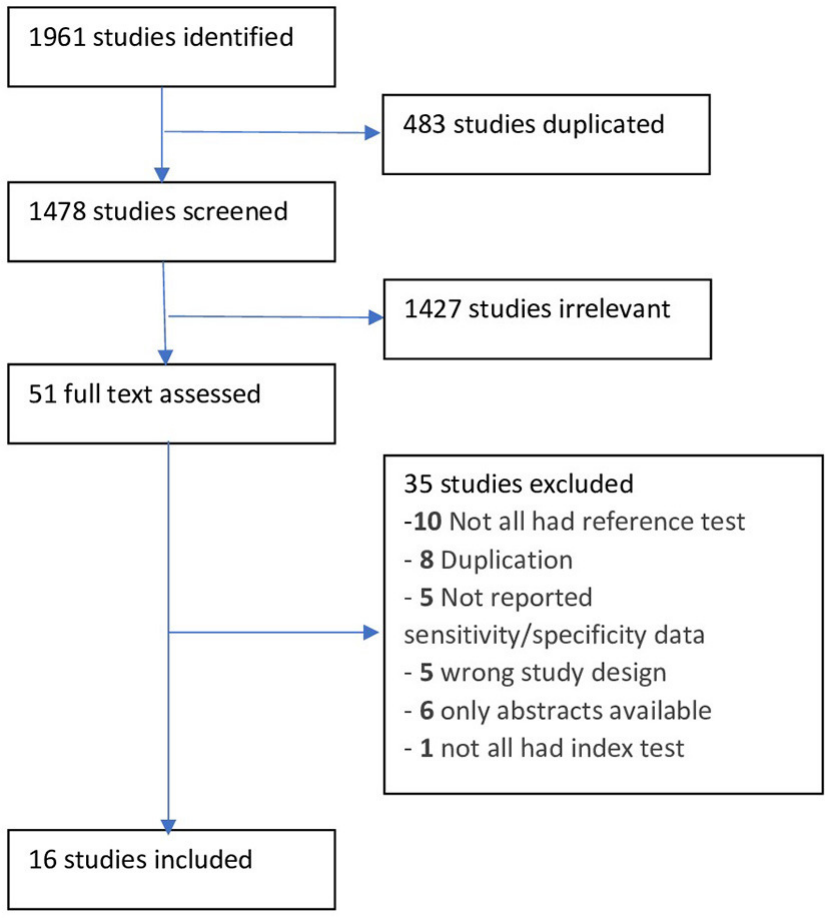
PRISMA flowchart.

Of the 1961 studies identified, 16 were included in the analysis (Figure 1) (15, 22, 24, 29–41). Most studies investigated the detection of AF by BP devices in office settings (*n* = 13), and only a few investigated AF detection by ABPM (*n* = 2) and HBPM (*n* = 1). Six (37.5%) studies used BP devices that were not clinically validated for BP measurements by international hypertension organisations (www.stridebp.org). The included studies investigated BP devices produced by Microlife (*n* = 11, [5 used Watch BP series]), Omron (*n* = 5), A&D (*n* = 1), and OSTAR Meditech Corp (*n* = 1) (Table 1). The BP devices used different algorithms to detect AF; the algorithms used by Omron were unclear despite our personal communication with the manufacturer (Supplementary Table S2A). In particular, the Heart Spectrum BP Monitor by OSTAR Meditech Corp used a substantially different method (i.e., fast Fourier transform analysis) to detect AF and was therefore not included in the meta-analyses (Supplementary Table S2A) (33). Different criteria to detect AF by the Heart Spectrum BP monitor (*n* = 29) were also investigated, and no single overall sensitivity (ranging from 90-100%) and specificity (ranging from 94–100%) was provided (33).

**Table 1:** Characteristics of included studies.

**Study**	**Country**	**Sponsor by BP device company?**	**Primary care or general population?**	**Participants**	**BP device tested and their AF detection algorithm**	**Reference test**	**Mean age (yrs)**	**Proportion with AF**	**No. of participants**	**Sensitivity**	**Specificity**
Office BP Chan (15)	Hong Kong SAR	No	Yes	Patients with HT or DM, excluded: pacemaker, and/or an implanted defibrillator	Microlife WatchBP Home A	12 lead ECG by cardiologist	67.2	0.01	5,969	0.81	0.97
AlAwwa (29)	Jordan	No	No	Patients with ESRD undergoing maintenance hemodialysis	Omron M6	12-lead ECG by cardiologist	57	0.08	227	0.83	0.94
Kearley (24)	UK	No	Yes	Aged 75 years or over, living at home; excluded: pacemaker, and/or an implanted defibrillator	Microlife WatchBP	12 lead ECG by cardiologists	79.7	0.08	999	0.94	0.9
Wiesel (41)	USA	No	No	Unselected outpatients in urban cardiology practise who had an ECG performed, excluded: pacemaker	Omron 712C automatic sphygmomanometer^*^	12-lead ECG	69	0.12	446	1	0.91
Wiesel (40)	USA	Yes	No	Patients aged >= 50 years; exclude: pacemakers or defibrillators	Microlife BP A200 Omron M6 Comfort (HEM-7223-E)^*^	12-lead ECG	74	0.16	183	1 (Microlife) 0.3 (Ormon)	0.92 (Microlife) 0.97 (Ormon)
Gandolfo (31)	Italy	No	No	Recent onset stroke or TIA patients, excluded: pacemakers or defibrillators	Microlife AFib BP3MQ1-2D	12-lead ECG by cardiologist	77.7	0.18	207	0.89	0.99
Lown (35)	UK	No	Yes	Aged>65 both with and without a diagnosis of AF; excluded: pacemaker	Microlife Watch BP	12-lead ECG by cardiologists	73.9	0.19	418	0.96	0.94
Marazzi (36)	Italy	No	No	Consecutive patients referred to hypertension clinic.	Microlife BP A200 Plus OMRON M6	12-lead ECG	67	0.2	503	0.92 (Microlife) 1 (Omron)	0.95 (Microlife) 0.94 (Omron)
Balanis (30)	Germany	Yes	No	older than 20 years old, exclusion: pacemakers	Omron BP785N (HEM-7321-Z)^*^	12-lead ECG	70.2	0.2	99	1	0.85
Wiesel (33)	USA	Yes	No	Unselected general cardiology outpatients, Excluded: pacemaker, and/or an implanted defibrillator	BP3MQ1-2D; Microlife USA^*^	12-lead ECG	73	0.23	405	0.97	0.88
Watanabe (38)	Japan	Yes	No	Patients with AF and cheque-up examinees	UA-1020; A&D	ECG	57.02	0.27	280	1	0.97
Stergiou (37)	Greece	No	No	Patients with AF, other arrthymia or sinus rhythm. Excluded: pacemaker, and/or an implanted defibrillator	Microlife BPA100 Plus	12-lead ECG	70.5	0.37	73	1	0.89
Kao (33)	Taiwan	No	No	patients with AF and without, Excluded: pacemaker, and/or an implanted defibrillator	The Heart Spectrum Blood Pressure Monitor (P2; OSTAR Meditech Corp.)^*^	12-lead ECG	67	0.53	62	0.9-1 (testing multiple methods)	0.94-1 (testing multiple methods)
HBPM Wiesel (39)	USA	Yes	No	Patients with HT, DM, CHF, stroke; excluded: pacemakers or implantable defibrillators	Microlife BPM BP3MQ1-2D^*^	Event recorder when conducting HBPM	67	0.11	139	1	0.9
ABPM Huppertz (32)	UK	Yes	No	An implanted pacemaker for sick sinus syndrome and previously documented AF or AHRE, excluded: diagnosed with permanent AF, or (iv) had a VVI pacemaker	Microlife WatchBPO3 AFIB	Implantable pacemaker	71.6	0.08	48	0.76	0.81
Kollias (34)	Greece	Yes	No	Patients referred to a Hypertension Clinic with symptoms suggesting arrhythmias or with stroke or AF history, exclusion: pacemaker implantation	Microlife WatchBP O3 Afib	24-h Holter monitoring	70.55	0.21	100	0.93	0.87

* Not listed as preferred devices or validated devices on StrideBP.org.

AF, atrial fibrillation; BP, blood pressure; DM, diabetes mellitus; ECG, electrocardiogram; ESRD, end-stage renal disease; HT, hypertension; TIA, transient ischemic attack; UK, United Kingdom.

Each included study recruited 48–5969 participants, and a total of 10,158 participants were included in this review. Although only 7.3% of the participants showed AF, the prevalence of AF was high (≥15%) in most of the studies (*n* = 10) (Supplementary Table S2B; Table 1). The mean age of participants was 68.9 years. Most of the participants were female (50.9%), had HT (77.3%), and had no diabetes mellitus (62%) (Supplementary Table S2B).

**Figure 2:**
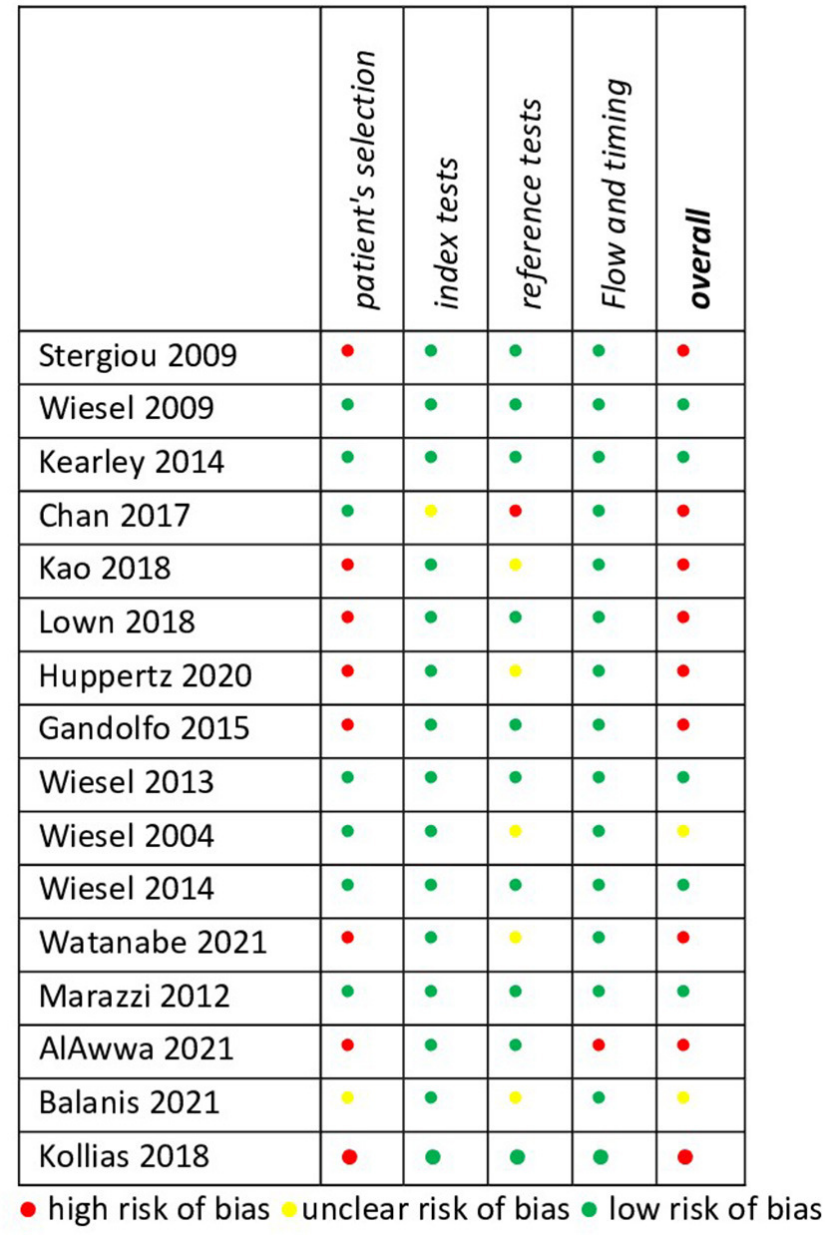
Quality assessment.

We identified only five studies (31.3%) with a low risk of bias because many studies employed the case-control design or were recruited from highly specialised settings, limiting their external validity (Figure 2). Further, only three studies (18.8%) were conducted in primary care settings.

‘

### Office BP

**Figure 3:**
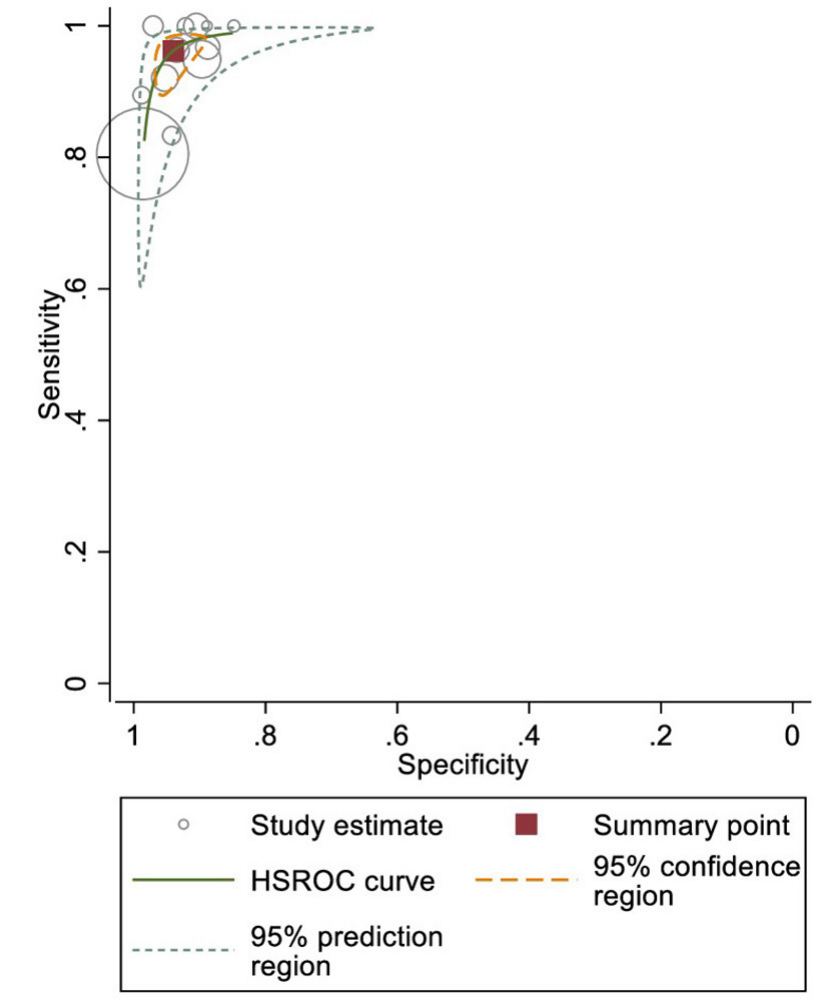
Sensitivity and specificity of office blood pressure (BP) devices to detect atrial fibrillation (AF).

When used in the office, BP devices had a sensitivity of 96.2% (95% confidence interval [CI]: 92.3–98.2%) and specificity of 94.0% (95% CI: 90.9–96.1%) to detect AF; the results were homogeneous (prediction ellipse area: 4.8%, MOR for sensitivity: 2.37, MOR for specificity: 2.02; Figure 3). Instead of using ≥2 BP measurements to detect AF (i.e., ≥2 AF detected out of two or three consecutive BP measurements), a few studies reported using only one BP measurement to detect AF (36–38, 40). Although most of these studies reported similar sensitivity and specificity, specificity may be reduced in studies that detected AF using any positive result among three consecutive BP measurements (Supplementary Figures S3A,S3B; meta-analysis was not conducted due to high heterogeneity and inadequate number of studies.) (36–38, 40). Stergiou et al. and Watanabe et al. directly compared the effect of different numbers of BP measurements and found better sensitivity and specificity when the “majority rule” (i.e., ≥2 positive readings out of two or three measurements) were used (37, 38).

Other subgroup and sensitivity analyses, including different BP devices and only low-risk-of-bias studies, yielded comparable sensitivities and specificities (Supplementary Table S4). Although the Omron device subgroup had wide 95% CIs, this can be explained by an outlier (Wiesel, Arbesfeld, and Schechter 2014) reporting a sensitivity of 30% (40).

### Out-of-office BP

The limited number of studies (*n* = 3) precluded a meta-analysis for the out-of-office BP subgroup analysis. All relevant studies used Microlife devices (Microlife WatchBP O3 Afib [ABPM] and Microlife BPM BP3MQ1-2D [HBPM]).

**Figure 4:**
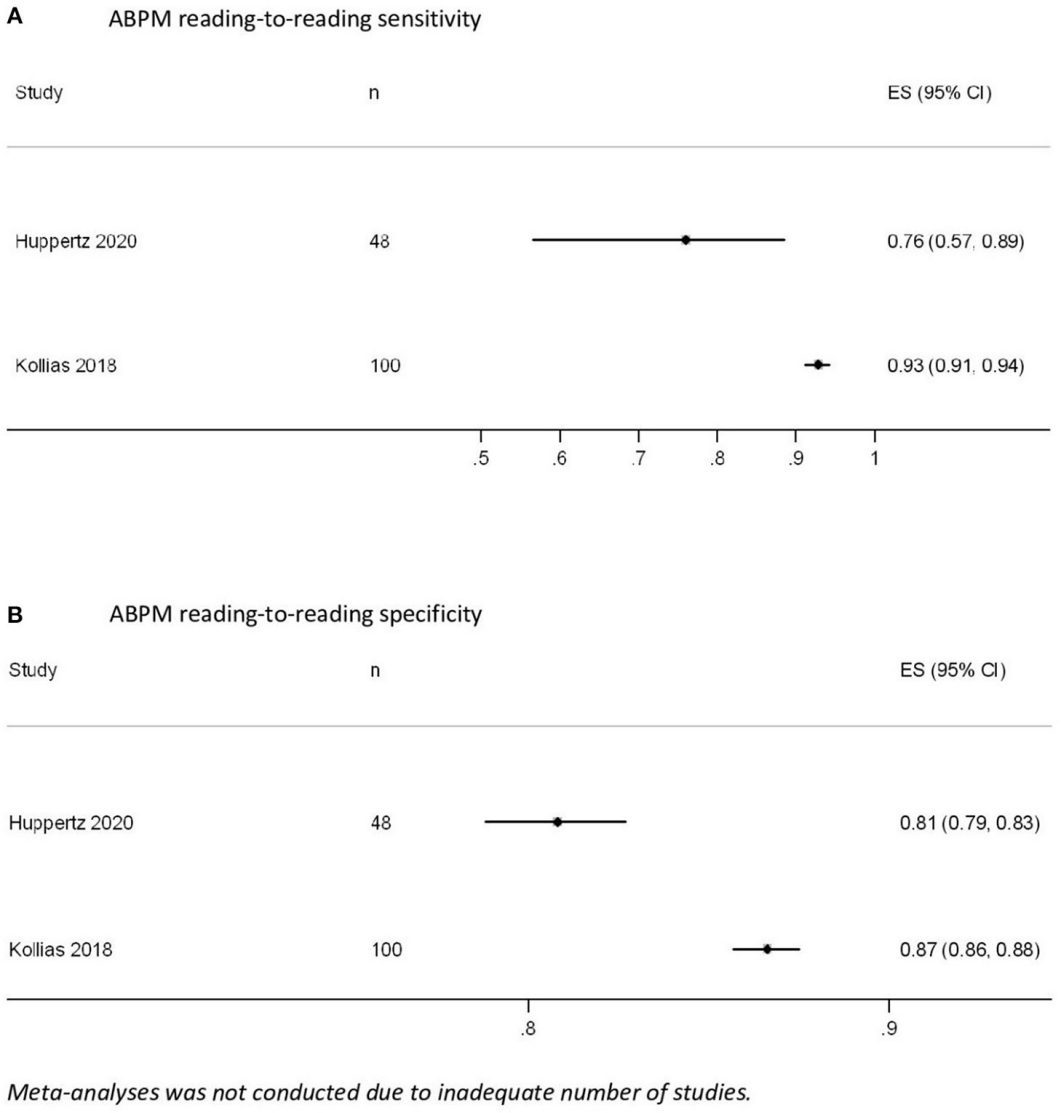
Ambulatory blood pressure measurement (ABPM) reading-to-reading **(A)** sensitivity and **(B)** specificity.

Huppertz et al. (*n* = 48, compared to implanted pacemaker) and Kollias et al. (*n* = 100, compared to 24-h Holter) reported sensitivity and specificity of each ABPM reading, which were 76–93% and 81–87%, respectively (Figure 4) (32, 34). Kollias et al. further suggested the use of ≥79 and ≥26% of positive readings on ABPM to detect persistent AF (with 100% sensitivity and specificity) and pAF (as defined by AF of duration >30 s; with 100% sensitivity and 85% specificity), respectively (34). Furthermore, a lower threshold (i.e., ≥15%) can be considered for high-risk patients to detect AF of any duration (with 90% sensitivity and 77% specificity) (34). Both of these studies had limited external validity for AF screening in the general population or in primary care because they only recruited patients referred for specialist care or with pacemakers (32, 34).

Only Wisel et al. (*n* = 139, compared to event recorders) reported the accuracy of HBPM for detecting AF or pAF over a 30-day period. AF was detected over three consecutive BP measurements using a majority rule (≥2 out of three readings) and confirmed on the fourth reading. The sensitivity, specificity, positive predictive value, and negative predictive value were 100% (95% CI: 76.84–100%), 89.6% (95% CI: 82.87–94.35%), 51.85% (95%CI: 39.16–64.31%), and 100% respectively (39).

## Discussion

### Main findings

Consistent with the data of previous meta-analyses, our study showed high sensitivity (96.2%) and specificity (94%) of office BP devices in detecting AF (14, 17–20). Further, similar to previous reports, our study also did not recommend employing only one positive result among consecutive office BP measurements to detect AF because of inferior specificity (Supplementary Figure S3) (14, 18). This systematic review for the first time, to the best of our knowledge, lists all existing algorithms employed for detecting AF in different devices (Supplementary Table S2A). Despite different measurement algorithms, the devices from Microlife and Omron had comparable sensitivities and specificities (Supplementary Table S4). We also summarised the results from existing ABPM and HBPM studies that may guide clinical management.

### Clinical implications

Validated office BP devices can be implemented clinically to screen for AF. However, as suggested by international guidelines, screening should be reserved for high-risk populations (i.e., age ≥65 years) because, despite high sensitivity and specificity, the positive predictive values are highly dependent on the prevalence of AF (8–10). For instance, at a prevalence of 1 and 10%, the positive predictive values were 13.94 and 64.05%, respectively (Supplementary Table S5). Screening in low-risk populations would result in a large number of patients with false-positive results, which are potentially harmful and expensive. False-positive results can be due to other arrythmias and positive results should therefore be confirmed by 12-lead ECG (for suspected AF) and/or 24-h Holter (for suspected pAF) prior to any treatment (32, 34). Furthermore, anti-coagulant treatments are often not indicated for low-risk AF patients (42). Clinicians should be alerted that only a few BP devices have been tested for AF detection. Although a list of validated machines for BP measurements was produced by international hypertension societies, similar lists for AF detection are lacking (23). To facilitate clinical recommendations, BP device manufacturers should disclose their AF detection algorithm; currently the AF detection strategy of the Omron devices is unclear (Supplementary Table S2A). Before the recommendation lists become available, clinicians may refer to our results (Supplementary Table S2; Table 1) or search for relevant validation studies to identify suitable devices. Newer BP devices incorporating single-lead ECG are now available and may facilitate AF detection. However, our search did not identify any relevant validation studies and these devices need to be validated prior to routine implementation in clinical practise. Also, when screening for AF, the “majority rule” (i.e., ≥2 positive results out of two or three consecutive BP measurements) appeared more specific and may reduce false-positive results. This is also clinically relevant because multiple office BP measurements during every office visit are recommended by international guidelines to obtain accurate BP values (43).

### Research implications

A threshold of number/proportion of positive results to detect AF or pAF on HBPM and ABPM is required. Although studies consistently report high sensitivity and specificity of individual HBPM/ABPM readings, false-positive results are common and expected due to repeated (on average 48–72 measurements per 24-h ABPM) measurements. For example, at a specificity of approximately 85%, one or two false-positive results are expected from 10 measurements. Kollias et al. observed that 13.4% of ABPM readings were false-positives in non-AF participants (34), and were due to body movements and other arrhythmias (32, 34). Although Kollias et al. (*n* = 100) proposed several thresholds to detect pAF and AF, these should be externally validated in large cohorts, preferably in primary care populations (34). Similar studies are lacking for HBPMs. Furthermore, newer devices (from A&D and OSTAR Meditech Corp.) use different novel algorithms to detect AF, but were only investigated in one study, and hence, require further external validation. Randomised controlled trials (RCTs) may be conducted to investigate the utility of these BP devices (with or without other AF detection methods) in primary care settings in improving detection of AF, increasing subsequent anticoagulation treatments, reducing cardiovascular morbidity and mortality, and being cost effective. Currently, evidence is conflicting and no RCTs have investigated the effect of AF screening by BP devices on mortality. For instance, although Uittenbogaart et al. found no improvement in AF detection by implementation of BP devices (possibly due to good AF detection rates in usual care), another RCT found that screening of AF with HBPM and ECG patches can increase AF detection by 10-fold and increase anticoagulation use (11, 44). Finally, cost-effectiveness studies conducted in the United Kingdom have suggested that AF screening by office BP can be cost-saving (45); however, cost-effectiveness results will vary across different countries.

### Strengths and limitations

Our systematic review and meta-analysis were registered, contained the largest number of studies for analysis, and included and discussed HBPM and ABPM studies (14, 17–20). Our results concerning office BP were homogenous and were analysed using the recommended statistical methods (21). This review, in a first, lists and investigates different AF detection algorithms used by different BP devices and provides important clinical guidance. Relevant subgroup and sensitivity analyses showed similar results, confirming that our results are robust. This has facilitated the development of practical clinical and research recommendations.

Our study had some limitations. First, only English literature was searched because of the lack of translators (this study was entirely self-funded). However, because many HT studies in other languages usually publish their abstracts in English, it is unlikely that a significant amount of literature was excluded. We did not exclude any studies due to language limitations. Furthermore, despite being stated in our registered protocol, meta-analyses for some subgroups could not be performed because of an inadequate number of studies. For example, Microlife Watch BP was only used by three office BP studies. Similarly, definite conclusions could not be drawn for ABPM (*n* = 2) and HBPM (*n* = 1) because of the small number of studies. Although some studies were conducted in primary care (where screening for AF is likely to take place, *n* = 3) and were considered to have a low risk of bias (*n* = 5), all subgroup or sensitivity analyses had similar results. Lastly, the estimation of sensitivity and specificity is affected by the prevalence of disease, but most studies had a higher prevalence of AF than that of the general population (Supplementary Table S2; Table 1) (46). Although this may limit the external validity of our results, the sensitivity analyses using only studies with a high prevalence of AF yielded similar results (Supplementary Table S4).

### Perspective

Our study showed that validated office BP devices have high sensitivity and specificity for AF detection, and may be used by clinicians for AF detection, particularly in high-risk populations using correct techniques. In future, RCTs are needed to determine whether AF screening using office BP devices can reduce strokes and mortality. In addition, more studies are needed to guide the use of HBPM and ABPM in detecting pAF or AF.

## Data availability statement

The original contributions presented in the study are included in the article/Supplementary material, further inquiries can be directed to the corresponding author/s.

## Author contributions

EL conceptualised the study. EL and ET screened and selected eligible studies, conducted data extraction, data analysis, and drafted the manuscript. C-PY designed and conducted the literature search. All authors contributed to the article and approved the submitted version.

## Conflict of interest

The authors declare that the research was conducted in the absence of any commercial or financial relationships that could be construed as a potential conflict of interest.

## Publisher's note

All claims expressed in this article are solely those of the authors and do not necessarily represent those of their affiliated organisations, or those of the publisher, the editors and the reviewers. Any product that may be evaluated in this article, or claim that may be made by its manufacturer, is not guaranteed or endorsed by the publisher.

## Supplementary material

The Supplementary Material for this article can be found online at: https://www.frontiersin.org/articles/10.3389/fcvm.2022.956542/full#supplementary-material

## Abbreviations

ABPM: ambulatory blood pressure monitoring
AF: atrial fibrillation
BP: blood pressure
HBPM: home blood pressure monitoring
HT: hypertension
pAF: paroxysmal AF.
