# Supplementary material for: Sensitivity and specificity of automated blood pressure devices to detect atrial fibrillation: A systematic review and meta-analysis of diagnostic accuracy
Source: Front Cardiovasc Med. 2022 Aug 12;9:956542. doi: 10.3389/fcvm.2022.956542 (PMC9411860; doi:10.3389/fcvm.2022.956542)
Supplement: Supplementary file 1 [file Data_Sheet_1.docx]

Supplementary table S1. Detailed search strategy

Search strategy for Ovid MEDLINE:

| 1 | Arrhythmias, Cardiac/ or Atrial Fibrillation/ |
| --- | --- |
| 2 | (Cardiac Arrhythmia* or Heart arrhythmia* or Atrial Fibrillation or Afib or AF).ti,ab. |
| 3 | Blood Pressure Monitoring, Ambulatory/ or Blood Pressure Monitors/ or Blood Pressure Determination/ |
| 4 | ((Ambulatory or Automated or Automatic or Oscillometric or clinic or clinical or Home or Office) adj (blood pressure or BP) adj (Machine* or Monitor* or Measure* or Measuring or Device* or Determination)).ti,ab. |
| 5 | (ABPM or AOBP or Microlife or WatchBP or OMRON).ti,ab. |
| 6 | 1 or 2 |
| 7 | 3 or 4 or 5 |
| 8 | "Sensitivity and Specificity"/ or Early Diagnosis/ |
| 9 | (Accurate or Accuracy or Detect* or Detection or Diagnose* or Diagnosis or Diagnostic or Sensitivity or Specificity or Screening or Valid*).ti,ab. |
| 10 | 8 or 9 |
| 11 | 6 and 7 and 10 |

The same group of keywords and subject headings were used for other databases.

Only keywords were used for Web of Science and Scopus.

Supplementary table S2a algorithm to detect AF by different BP devices described by included studies

| **Manufacturer** | **Model** | **Algorithm** |
| --- | --- | --- |
| Microlife | Microlife BPA100 Plus, BP3MQ1-2D, Microlife Watch BP, Microlife BP A200 Plus | The average time interval of the last 10 beats, during deflation, is calculated and intervals that are 25% shorter or longer than that of the average are discarded. The mean of the remaining intervals is calculated with its s.d., and an AF diagnosis is made, if the s.d. per mean ratio is>0.06 |
| Omron* | Omron 712C automatic sphygmomanometer,  Omron M6 | irregularity index>0.066^4,8^ |
|  | Omron M6 | irregular heartbeat detector measuring the last ten pulse intervals during cuff deflation, and then calculating the mean and standard deviation of the intervals. An irregularity index is defined as the standard deviation divided by the mean of the time intervals. To reduce the effect of premature beats on the irregularity index, a cut-off value of 25% was chosen, so that each of the ten pulse beat intervals that is 25% greater than or 25%less than the mean time interval was deleted. The remaining time intervals are used to calculate the irregularity index. If the irregularity index surpasses a threshold value of 0.06, the rhythm is considered irregular^2^ |
|  | Omron BP785N (HEM-7321-Z) | indicate an irregular heartbeat (IHB) that is detected when there is a deviation of more than 25% from average^9^ |
| A&D | UA-1020 | Irregular pulse peak (IPP) 15 was defined as follows: \|interval of pulse peak - the average of the interval of the pulse peak\| ≥ the average of the interval of the pulse peak × 15%. Irregular heartbeat (IHB) was defined as follows: beats of IPP ≥ total pulse × 20%. |
| OSTAR Meditech Corp | The Heart Spectrum blood pressure monitor | The Heart Spectrum Blood Pressure Monitor calculated the heart spectrum results via FFT analysis for the diagnosis of AF. |

*Discrepancies on algorithm to detect AF as presented by different included studies (as shown). The reviewers contacted Omron, whose staff refused to disclose algorithms because these were considered confidential

Supplementary table S2b demographics of included participants

|  | Value | No of studies reporting this parameter |
| --- | --- | --- |
| Overall number of participants | 10158 | N/A |
| Proportion with AF | 7.3% | 16 |
| Weighted mean age (years) | 68.94 | 16 |
| Proportion of males | 49.1% | 14 |
| Proportion with hypertension | 77.3% | 10 |
| Proportion with diabetes | 38% | 10 |
| Proportion with cardiovascular disease | 8.8% | 8 |
| Mean Systolic BP (mmHg) | 126.4 | 4 |

**Included studies**

1. Chan P-H, Wong C-K, Pun L, et al. Diagnostic performance of an automatic blood pressure measurement device, Microlife WatchBP Home A, for atrial fibrillation screening in a real-world primary care setting. *BMJ open* 2017; **7**(6): e013685.

2. AlAwwa I, Saleh A, Wahbeh AM, et al. Accuracy and feasibility of portable blood pressure monitoring in the detection of atrial fibrillation in hemodialysis patients. *Reviews in Cardiovascular Medicine* 2021; **22**(1).

3. Kearley K, Selwood M, Van den Bruel A, et al. Triage tests for identifying atrial fibrillation in primary care: a diagnostic accuracy study comparing single-lead ECG and modified BP monitors. *BMJ open* 2014; **4**(5): e004565.

4. Wiesel J, Wiesel D, Suri R, Messineo FC. The use of a modified sphygmomanometer to detect atrial fibrillation in outpatients. *Pacing and clinical electrophysiology* 2004; **27**(5): 639-43.

5. Wiesel J, Arbesfeld B, Schechter D. Comparison of the Microlife blood pressure monitor with the Omron blood pressure monitor for detecting atrial fibrillation. *The American journal of cardiology* 2014; **114**(7): 1046-8.

6. Gandolfo C, Balestrino M, Bruno C, Finocchi C, Reale N. Validation of a simple method for atrial fibrillation screening in patients with stroke. *Neurological sciences* 2015; **36**(9): 1675-8.

7. Lown M, Yue AM, Shah BN, et al. Screening for atrial fibrillation using economical and accurate technology (from the safety study). *The American Journal of Cardiology* 2018; **122**(8): 1339-44.

8. Marazzi G, Iellamo F, Volterrani M, et al. Comparison of Microlife BP A200 Plus and Omron M6 blood pressure monitors to detect atrial fibrillation in hypertensive patients. *Advances in therapy* 2012; **29**(1): 64-70.

9. Balanis T, Sanner B. Detection of Atrial Fibrillation Using a Home Blood Pressure Monitor. *Vascular Health and Risk Management* 2021; **17**: 407.

10. Wiesel J, Fitzig L, Herschman Y, Messineo FC. Detection of atrial fibrillation using a modified microlife blood pressure monitor. *American journal of hypertension* 2009; **22**(8): 848-52.

11. Watanabe T, Tomitani N, Yasui N, Kabutoya T, Hoshide S, Kario K. Assessment of a new algorithm to detect atrial fibrillation in home blood pressure monitoring device among healthy adults and patients with atrial fibrillation. *The Journal of Clinical Hypertension* 2021; **23**(5): 1085-8.

12. Stergiou G, Karpettas N, Protogerou A, Nasothimiou E, Kyriakidis M. Diagnostic accuracy of a home blood pressure monitor to detect atrial fibrillation. *Journal of human hypertension* 2009; **23**(10): 654-8.

13. Kao W-F, Hou S-K, Huang C-Y, Chao C-C, Cheng C-C, Chen Y-J. Assessment of the clinical efficacy of the heart spectrum blood pressure monitor for diagnosis of atrial fibrillation: An unblinded clinical trial. *PloS one* 2018; **13**(6): e0198852.

14. Wiesel J, Abraham S, Messineo FC. Screening for asymptomatic atrial fibrillation while monitoring the blood pressure at home: trial of regular versus irregular pulse for prevention of stroke (TRIPPS 2.0). *The American journal of cardiology* 2013; **111**(11): 1598-601.

15. Huppertz N, Lip GY, Lane DA. Validation of the modified Microlife blood pressure monitor in patients with paroxysmal atrial fibrillation. *Clinical Research in Cardiology* 2020; **109**(7): 802-9.

16. Kollias A, Destounis A, Kalogeropoulos P, Kyriakoulis KG, Ntineri A, Stergiou GS. Atrial fibrillation detection during 24-hour ambulatory blood pressure monitoring: comparison with 24-hour electrocardiography. *Hypertension* 2018; **72**(1): 110-5.

Supplementary figure S3a. Sensitivity of office BP to detect AF when only 1 BP measurement is used (i.e. BP was only measured 1 time or 1 positive reading out of a few consecutive readings). Meta-analysis was not performed due to inadequate numbers of studies

** Wiesel 2014a/b and Stergiou 2009a/b are from the same study. Watanabe2021 used a different algorithm to define AF from other studies*

Supplementary table S3b Specificity of office BP to detect AF when only 1 BP measurement is used (i.e. BP was only measured 1 time or 1 positive reading out of a few consecutive readings). Meta-analysis was not performed due to inadequate numbers of studies

** Wiesel 2014a/b and Stergiou 2009a/b are from the same study. Watanabe2021 used a different algorithm to define AF from other studies*

Supplementary table S4a summary of subgroup/sensitivity analyses

|  | **Sensitivity** | **95%CI** | **Specificity** | **95%CI** |  |
| --- | --- | --- | --- | --- | --- |
| Main results | 96.2% | 92.3-98.2% | 94% | 90.9-96.1% | Figure 3 |
| Microlife devices only | 94.4% | 89.8-97% | 94.5% | 90.5-96.8% | S4b |
| Omron devices only | 99.7% | 33.1-100% | 93.3% | 89.7-95.7% | S4c |
| Low-risk of bias studies only | 95.4% | 91.6-97.5% | 91.6% | 88.3-94% | S4d |
| Specialist centers only | 98.1% | 91.8-99.6% | 93.4% | 90.1-95.6% | S4e |
| Prevalence of AF ≥15% | 97.3% | 93-99% | 93.6% | 89.9-96% | S4f |
| prevalence of AF ≥20% | 98.4% | 89.8-99.8% | 92.4% | 87.2-95.6% | S4g |
| Funded by BP device manufacturers** | 97.3% | 93.5-98.9% | 91.8% | 85.9-95.4% | S4h |
| Validated BP devices only (as defined by StrideBP.org) | 95.6% | 89.7-98.2% | 94.8% | 91.6-96.8% | S4i |

*CI – confidence interval

** Stata does not accept “0” cells in this models and these cells were replaced by “1”

Supplementary figure S4b Office BP to detect AF, Subgroup: only included Microlife devices


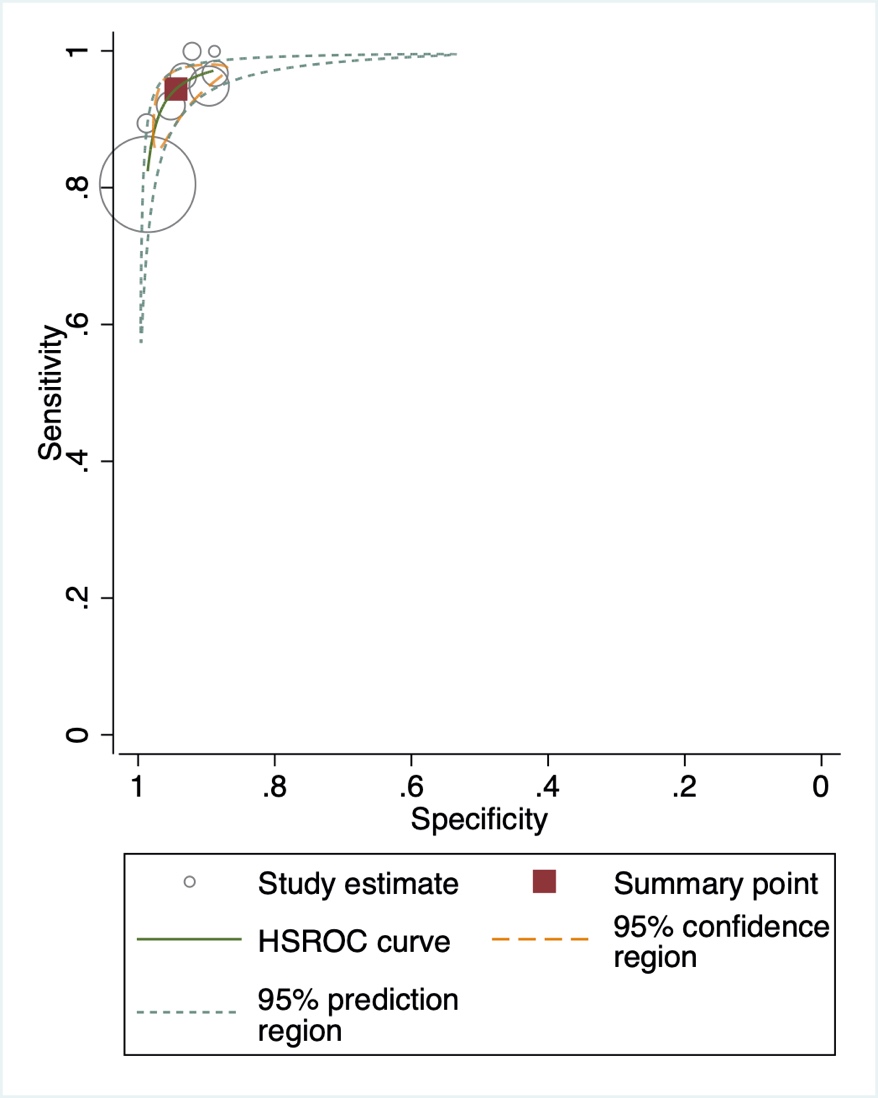


Sensitivity 94.4% (95%CI: 89.8-97%); Specificity 94.5% (95%CI: 90.5-96.8%)

*Proportion of prediction region to area under ROC curve = 6.2%, median odds ratio: sensitivity 1.98, specificity 2.12

**each circle represents included studies and its size represents the sample size, HSROC: hierarchical summary receiver operating characteristic

Supplementary figure S4c Office BP to detect AF, Subgroup: only included OMRON devices

Sensitivity 99.7% (95%CI: 33.1-100%), specificity 93.3% (95%CI: 89.7-95.7%)

*Proportion of prediction region to area under ROC curve = N/A (‘R’ gave error message), median odds ratio: sensitivity 121.93, specificity 1.54

**each circle represents included studies and its size represents the sample size, HSROC: hierarchical summary receiver operating characteristic

Supplementary figure S4d Office BP to detect AF, Subgroup: low risk of bias only

Sensitivity: 95.4% (95%CI: 91.6-97.5%), specificity 91.6% (95%CI: 88.3-94%)

*Proportion of prediction region to area under ROC curve = 11.3%, median odds ratio: sensitivity 1.35, specificity 1.35

**each circle represents included studies and its size represents the sample size, HSROC: hierarchical summary receiver operating characteristic

Supplementary figure S4e. Office BP to detect AF, Subgroup: only included studies conducted in specialist centres or hospitals

Sensitivity: 98.1% (95%CI: 91.8-99.6%), specificity: 93.4% (90.1-95.6%)

*Proportion of prediction region to area under ROC curve = 9%, median odds ratio: sensitivity 3.95, specificity 1.75

**each circle represents included studies and its size represents the sample size, HSROC: hierarchical summary receiver operating characteristic

Supplementary figure S4f. Office BP to detect AF, Subgroup: only included studies with prevalence of AF ≥15%

Sensitivity 97.3% (95%CI: 93-99%), specificity 93.6% (95%CI: 89.9-96%)

*Proportion of prediction region to area under ROC curve = 5.9%, median odds ratio: sensitivity 2.17, specificity 1.83

**each circle represents included studies and its size represents the sample size, HSROC: hierarchical summary receiver operating characteristic

Supplementary figure S4g. Office BP to detect AF, Subgroup: only included studies with prevalence of AF ≥20%

Sensitivity 98.4% (95%CI: 89.8-99.8%), specificity 92.4% (95%CI: 87.2-95.6%)

*Proportion of prediction region to area under ROC curve = 34.9%, median odds ratio: sensitivity 2.92, specificity 1.72

**each circle represents included studies and its size represents the sample size, HSROC: hierarchical summary receiver operating characteristic

Supplementary figure S4h Office BP to detect AF, Subgroup: only included studies supported by BP device manufacturers

Sensitivity: 97.3% (95%CI: 93.5-98.9%), specificity 91.8% (95%CI: 85.9-95.4%)

*Proportion of prediction region to area under ROC curve = 35.1%, median odds ratio: sensitivity 1.45, specificity 1.69

**each circle represents included studies and its size represents the sample size, HSROC: hierarchical summary receiver operating characteristic

Supplementary figure S4i Office BP to detect AF, Subgroup: only validated BP devices by StrideBP.org


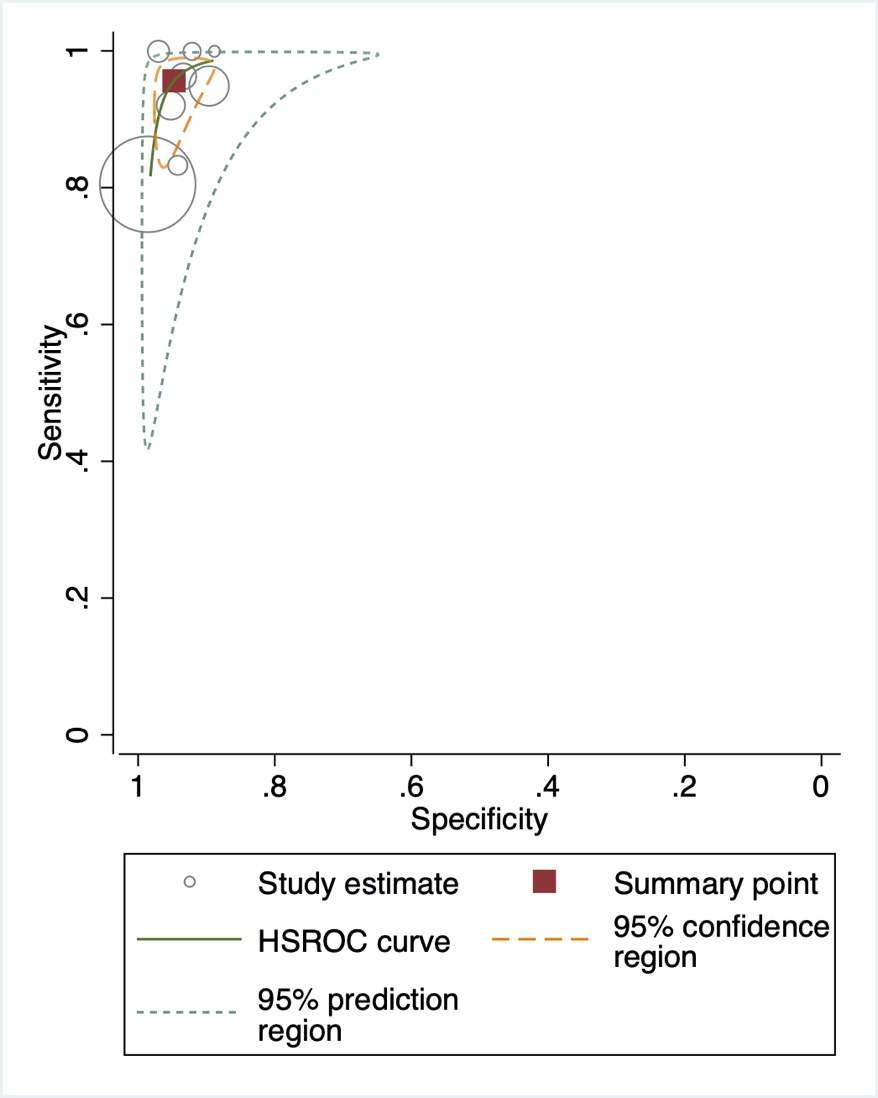


Sensitivity 95.6% (95%CI: 89.7-98.2%), specificity 94.8% (95%CI: 91.6-96.8%)

*Proportion of prediction region to area under ROC curve = 6.9%, median odds ratio: sensitivity 2.50, specificity 1.89

**each circle represents included studies and its size represents the sample size, HSROC: hierarchical summary receiver operating characteristic

Supplementary table S5 – positive predictive value at different prevalence of AF using our results (sensitivity 96.2%, specificity 94%)

| Prevalence | Positive Predictive Values (PPV) |
| --- | --- |
| 0.1% | 1.58% |
| 0.5% | 7.46% |
| 1% | 13.94% |
| 5% | 45.77% |
| 10% | 64.05% |
| 15% | 73.89% |
| 20% | 80.03% |
